# Assessment of Knowledge, Attitude, and Practice of Security and Safety Workers Toward the COVID-19 Pandemic: A Cross-Sectional Study

**DOI:** 10.3389/fpubh.2021.631717

**Published:** 2021-04-28

**Authors:** Maha M. AlRasheed, Abdullah M. Alsugair, Hala F. Almarzouqi, Gadah K. Alonazi, Fadilah S. Aleanizy, Fulwah Y. Alqahtani, Gamal A. Shazly, Fowad Khurshid

**Affiliations:** ^1^Department of Clinical Pharmacy, College of Pharmacy, King Saud University, Riyadh, Saudi Arabia; ^2^Department of Civil Engineering, College of Engineering, King Saud University, Riyadh, Saudi Arabia; ^3^Department of Pharmaceutics, College of Pharmacy, King Saud University, Riyadh, Saudi Arabia; ^4^Department of Industrial Pharmacy, Faculty of Pharmacy, Assiut University, Assiut, Egypt

**Keywords:** COVID-19, pandemic, attitudes, practices, worker

## Abstract

**Aim:** This study aimed to assess the knowledge, attitude, and practice (KAP) of security and safety workers toward the COVID-19 pandemic in Saudi Arabia.

**Methods:** A cross-sectional survey was conducted between April and July 2020 using a self-developed structured questionnaire that was randomly distributed online among security and safety employees in government or private sectors.

**Results:** Among the 712 participants, 53.9% were female and the respondents' mean age was 39.43 years. Television was chosen as the most reliable source of information by 75.0% of the participants. Most of the respondents had a sufficient knowledge about the COVID-19 pandemic, as the majority of them answered the knowledge questions correctly. The significant predictors for their knowledge were their educational level, age, marital status, parenthood status, and employment sector (private or government). Our study revealed an overall 98.6% positive attitude of safety and security workers toward COVID-19. Majority of the respondents were following good and safe COVID-19 prevention practices.

**Conclusion:** High level of knowledge was reflected in both the attitude and practice of the participants toward the COVID-19 pandemic.

## Introduction

At the end of December 2019, a novel virus, Severe Acute Respiratory Syndrome-Coronavirus-2 (SARS-CoV-2), termed COVID-19, surfaced in Wuhan, China, and has since spread widely across the globe with a substantial clinical impact (1). Based on the currently available information, COVID-19 is a highly contagious disease which can spread through human-to-human transmission. Its primary reported manifestation includes fever, fatigue, dry cough, myalgia anorexia, and sore throat (1, 2). Global concerns about the spread of the virus have risen due to its high transmissibility primarily as respiratory droplet discharge from the mouth or nose through coughing or sneezing (2). It has also been reported that the coronavirus is found in the tongue, mouth floor, and salivary secretions of affected patients, hence, highlighting the potential diagnostic capability of human saliva for the early detection of COVID-19 (3). Early and rapid findings through saliva can help to control the spread of the disease as well as further outbreaks of contagious viruses (3). A systematic review of COVID-19 patients has revealed that individuals with hypertension, diabetes, cardiovascular, and respiratory system diseases belong to the most vulnerable groups (4). Recently, the U.S. Food and Drug administration approved the antiviral drug Remdesivir to be the first and only approved COVID-19 treatment to date (5). At present, a couple of vaccines have been approved to be used in different countries so far. Therefore, to minimize the spread of the virus, preventive measures as recommended by competent authorities are of utmost significance. The ultimate decision on patient management and treatment should be made by the clinician to provide pertinent data in the patients' best interest (6), while adherence to the recommended measures is largely determined by the knowledge, attitude, and practice (KAP) of the community (7).

Saudi Arabia recorded the first confirmed case of COVID-19 on March 2, 2020 (8). With the continuous increase in the devastating numbers of new cases reported globally, on March 11, 2020, the World Health Organization (WHO) declared this outbreak a global pandemic (9). According to the WHO report, as of September 24, 2020, 31,664,104 confirmed cases and 972,221 confirmed deaths had been recorded in the world (10). By then, Saudi Arabia had also been seriously affected by the COVID-19 pandemic, with reports of 331,359 confirmed cases and 4,569 deaths (11). However, early precautionary procedures had been adopted by March 8, before the first case in Saudi Arabia was detected. These included the suspension of entry into KSA for Umrah as well as the closure of all schools and universities in both governmental and private sectors across the country thereby establishing provisions of alternatives to ensure continuity by distance learning. Also, travel to and from specific countries was initially banned on March 9. However, once the cases started to increase in neighboring countries, the government also barred entry to individuals without passports from the Gulf countries to avoid possible transmission emanating within the region in the immediate 2-week period. In particular, precaution was called for at this time requiring any person who has visited Iran recently or during the previous 2 weeks to contact the health authorities for SARS-CoV-2 testing. This was followed by the suspension of all international travel on March 14 for 2 weeks. The closure of malls, restaurants, and coffee shops followed 2 days later, leaving only the supermarkets and pharmacies open, and finally the closure of mosques on March 17. The number of cases continued to rise thereafter, triggering a partial curfew between 7 p.m. and 6 a.m. for 3 weeks starting on March 23. However, the continued culmination in the infection rate triggerred the extension of the curfew initially from 3 p.m. and later to a full day (12).

By the time we launched our data collection on April 30, 2020, the partial curfew had returned to 9 a.m. until 5 p.m. The chronological sequence of events during our study is summarized in Table 1.

**Table 1:** Sequence of events in fighting COVID-19 during the study period (13, 14).

**Period/ Date**	**Event**	**Consequences**
April 30, 2020	Launching of data collection.	
	Return of partial curfew from 9 a.m. until 5 p.m. (13).	
May 5, 2020	Imposition of penalties for violating any of the preventive measures (13).	Jail sentences of up to five years and fines up to SR500,000.00 (13).
May 7, 2020		Penalties imposed for any social gathering?, such as weddings, parties, and mass gatherings (13).
Eid Al-Fitr days (May 23–27)	24 h curfew imposed to prevent social gatherings (13).	
May 28, 2000	Curfew turned partial from 6 a.m.−3 p.m. (13).	
May 30, 2020	Curfew extended from 6 a.m.−8 p.m. (13).	Penalties for not wearing a face mask (13).
May 31, 2020	Friday prayers and all other prayers were permitted in the mosques (13).	
June 21, 2020	Curfews and penalties were lifted for not wearing a face mask, refusing to be checked for temperature when entering the public or private sector, or failure to adhere to social distancing rules (13).	Life then returned to normal in all cities of the Kingdom except for Makkah (13).
June 23, 2020	Minister of Health announces that Hajj would be limited in numbers and to those <65 years and free from chronic diseases under precautionary measures, with the understanding that pilgrims would be subject to home quarantine after performing the Hajj (13).	
July 2, 2020	Gatherings of a maximum of 50 people allowed with adherence to stipulated preventive measures (13).	Increase of the ICU beds in various regions of the Kingdom (13).
	End of data collection.	
July 30, 2020	On the Eid al-Adha day, the curfew was lifted, but emphasis on the adherence to the preventive measures remained in place (13).	
August 3, 2020	Hajj drew to a successful end with no record of additional coronavirus cases in the country (13).	Saudi Arabia records the lowest daily cases for three months, and high recovery rate >86% of the total cases recorded (13).
August 15, 2020	Saudi Minister of Education announces the resumption of online education for all Saudi public schools during the first 7 weeks of the new school year, as a precaution against the spread of the new coronavirus (13).	
	Minister announces online studies for theoretical courses and in attendance for practical courses for universities and technical training institutions (13).	
August 19, 2020	Ministry of Health announced a decrease in confirmed cases by 72 % and an increase in the recovery cases to 91.7% (13).	
September 13, 2020	Ministry announces a further decrease in the confirmed cases by 88 % and an increase in the recovery cases to 93% (13).	
October 4, 2020.	Permission to perform Umrah for citizens and residents inside the Kingdom, starting at a rate of 30% capacity (6,000 pilgrims/day) taking into account the precautionary measures (14).	

*The table summarizes the sequence of restrictions imposed by the officials and applicable penalties in line with the guidelines for combating the COVID-19 pandemic during the period of interest*.

Studies on knowledge, attitude, and practice (KAP) toward infectious diseases can collect information on what is known, believed, and done by a specific population and helps predict the outcomes. Moreover, such studies are important because the readiness to fight communicable diseases like COVID-19 begins with adequate knowledge, a positive attitude, and safe practices (7, 15). In contrast, it is believed that inadequate knowledge, negative attitudes, and unsafe practice toward infectious diseases lead to unnecessary chaos which facilitates the rapid spread of infections, thereby complicating efforts to preclude the spread of the disease (16). There is a paucity of KAP data on COVID-19 prevention among communities. At this critical moment of a rapid rise in cases, it becomes mandatory to conduct such a study to comprehend the community awareness and preparedness to fight against the disease, to facilitate putting in place evidence-based strategies in addressing the identified shortcomings in guiding future management of the disease. Moreover, it is not clear as to whether certain professional groups within a society may respond to the spread of the disease in a particular fashion, as compared to the rest of the community in general. Hence, the present study aimed to explore the KAP of security and safety workers in the prevention of the spread of COVID-19 in Saudi Arabia.

## Methodology

### Study Design and Setting

A cross-sectional survey was conducted between April and July 2020. All security and safety workers in the government and private sectors (hospitals, universities, ministries) in Saudi Arabia who are able to read and write and willing to participate irrespective of COVID-19 infection status were included in the study. The selection of the security and safety workers as study subjects was facilitated by the fact that the first two authors had an administrative assignment of managing a security and safety administration. Individuals who did not meet the above inclusion criteria were not eligible and were thus excluded from the study.

### Sample Size

The required sample size for this study was calculated using the Raosoft sample size calculator employing a margin of error of 5% and a confidence interval of 95% (17). An estimated sample size of 377 individuals was determined as an adequate number for the study. However, to minimize the errors, the sample size taken for this study was 712.

### Enrollment

The participants were enrolled between April 30, 2020 and July 2, 2020. Enrollment time was divided into six periods according to the precautionary measures at that time. The first period started from April 30, 2020 until May 6, 2020 observing partial curfew from 9 a.m. to 5 p.m. In the second period (May 7, 2020 until May 5, 2020), the curfew time stayed the same. However, penalties for any social gathering were imposed and a 24-h curfew for Eid Al-Fitr days was applied in the third period running from May 28, 2020 to May 30, 2020. These curfew times were changed to start at 6 a.m. and end at 8 p.m. and penalties were imposed for not wearing a face mask in the fifth period. In the last period covering June 21, 2020 until July 2, 2020, the curfew was lifted, but penalties were enforced for not wearing a face mask, refusal to have temperature checked on entering a public or private sector, as well as failing to adhere to the social distancing rules (12).

### Outcome Measures

The present study examined the level of knowledge, attitude, and proper practice toward COVID-19 prevention using gender, age, education level, marital status, and work sector as potential explanatory variables among the security and safety workers in Saudi Arabia.

### Study Questionnaire

A standardized (structured, pre-coded, and validated) questionnaire was developed, based on an extensive literature review of previously published literature (7, 15, 18) as well as recent available information from the World Health Organization (WHO) and Saudi Ministry of Health (MOH) official websites. The survey questionnaire was refined from validated questionnaires that had been previously used to address our objectives. The designed questionnaire was validated in two steps. First, the initial draft of the questionnaire was sent to a group of experts in related fields to reflect on the relativity, simplicity, and importance of the questions. Secondly, the structured questionnaire was validated by piloting the survey on a group of 12 participants to make sure that the survey would work properly. Suitable amendments were then made to develop the final questionnaire based on their feedback. The pilot study data were not included in the final analysis. Since it was not feasible to conduct a community-based sampling survey during this critical period, we decided to collect the data online. Hence, the survey was made available on the “Google Forms” online survey platform, which is considered user-friendly and easily accessible on different web browsers (19). Also, the published survey was promoted in social media platforms to reach the target sample size. The self-reported questionnaire was divided into four sections. The first section consisted of 12 questions that determined the respondent's perceived level of knowledge concerning COVID-19. The second section of the survey consisted of eight questions addressing the attitude of the respondents. The third section included seven statements determining their practice toward the disease, while the fourth section included items providing information about the demographic characteristics (age, gender, educational level, marital status, and workplace) of the respondents. The questionnaire was designed in English, subsequently translated into Arabic for the convenience and easy understanding of the participants, and pre-tested to ensure that it maintained its original meaning.

### Assessment of Knowledge, Attitude, and Practice

Knowledge was assessed by a three-item scale. A score of one point was given if the correct answer was chosen and zero in the case of a wrong answer or “do not know” response. The total knowledge score was calculated by adding the scores with a maximum obtainable score of 12 for each participant. Total knowledge score was expressed as mean (SD). Total knowledge score was categorized into two levels, poor knowledge (≤ 10), and good knowledge (>10). The attitude score was calculated as a continuous variable by adding the respondent's number of appropriate answers to eight questions. One point was allotted for each appropriate response (agree) that was considered as a positive attitude and zero was given for each “disagree” or uncertain response, which was considered as a negative attitude, with a maximum attainable score of six for each participant. The mean attitude score for each respondent was calculated by dividing the total attitude scores by eight. A score of ≥0.5 was taken as positive and <0.5 as negative attitude. The practice score was calculated as a continuous variable by adding the respondent's number of appropriate responses to seven questions. Two points were given for “Yes,” one point for “Sometimes,” and zero points were given for “No” with a maximum obtainable score of 14 for each participant. Mean practice score for each respondent was calculated by dividing the total practice score by seven. A score of ≥1 was rated as good and <1 as poor practice.

### Statistical Analysis

Descriptive analysis was conducted, and data were reported as percentage and frequency. Knowledge scores, attitudes, and practices of the respondents according to their demographic characteristics were compared by independent-samples *t*-test, one-way analysis of variance (ANOVA), or Chi-square test as appropriate. Binary logistic regression analyses were used to identify the factors associated with attitudes and practices. Spearman's rank correlation coefficient (*p* < 0.05) was used to evaluate the association between knowledge and attitude or practice. Data were analyzed using the Statistical Package for Social Sciences (IBM SPSS Statistics, version 26). The statistical significance level was accepted at *p* < 0.05 (two-sided).

### Ethics Approvals

Ethical approval for this study was obtained from the King Saud University College of Medicine Institutional Review Board. Since this study was conducted during the lockdown period, a Google survey was prepared with an online informed consent form on the first page carrying a brief explanation of the objectives and benefits of the study emphasizing the confidentiality of personal data and its sole use for the scientific work. Individuals were asked to complete the written consent prior to their contribution in the study.

## Results

The demographic descriptions of the respondents are summarized in Table 2. A total of 712 security and safety workers participated in the study. Of these, 53.9% were female. The respondents mean age was 39.4 years [standard deviation (SD): 7.97, range: 20–62]. Around 60.0% of the respondents possessed educational qualifications of a high school certificate or below, while 40.0% of them held a Bachelor's or higher degree. The majority of the participants (87.5%) engaged in field work and 89.4% were on duty during the period from February 2020 to May 2020 (at the time of the onset of COVID-19). Furthermore, the majority of them (71.9%) were Riyadh (the capital city) residents.

**Table 2:** Sociodemographic characteristics of participants (*N* = 712).

**Characteristics**		**Frequency (%)**
Gender	Male	328 (46.1)
	Female	384 (53.9)
Marital status	Married	512 (71.9)
	Others (never married, separated, divorced, widowed)	200 (28.1)
Children	0	172 (24.2)
	1 or more	540 (75.8)
Educational level	High school certificate and below	427 (60.0)
	Bachelor's degree or above	285 (40.0)
Age group	20–34 years	198 (27.8)
	35–49 years	417 (58.6)
	50–64 years	94 (13.2)
Do you have chronic disease	No	574 (80.6)
	Yes	138 (19.4)
Region	Riyadh	512 (71.9)
	Others (Eastern Province, Makkah, Madinah, Qassim, Tabuk, Northern Borders, Jawf, Jizan, Asir)	200 (28.1)
Workplace	Health facility	150 (21.1)
	Others (Educational, residential, commercial facilities)	562 (78.9)
You work in	Private Sector	261 (36.6)
	Government Sector	451 (63.3)
Years of experience	<10 years	463 (65.0)
	More than 10 years	249 (35.0)
The nature of your work	Field work	623 (87.5)
	Administrative work	89 (12.5)
Were you on duty from at the time of the onset of COVID-19	No	75 (10.5)
	Yes	637 (89.4)
Source of information^*^	Radio	153 (21.5)
	Television	534 (75.0)
	Posters	54 (7.6)
	News paper	165 (23.2)
	Seminars	106 (14.9)
	Neighbors and friends	23 (3.2)
	Internet and Social media	365 (51.3)
	Others	53 (7.4)

* *Multiple answers were possible*.

The main source of COVID-19 information reported by the participants was television, (75.0%) followed by internet and social media (51.3%), and by newspapers (23.3%). Results are summarized in Table 2.

### Respondents' Knowledge Level About COVID-19

Table 3 describes the current status of COVID-19 knowledgeability among security and safety workers in Saudi Arabia. The mean total knowledge score was 10.6 ± 1.4. A total of 435 (61.1%) respondents showed good knowledge, while 277 (38.9%) exhibited the opposite. Poor knowledge was more apparent in response to questions related to the symptoms of the disease, availability of certified treatments and vaccines, transmission, preventive measures, and ways of protection from COVID-19 in which the rates of incorrect responses were 47.1, 20.8, 22.4, 44.7, and 28.5%, respectively.

**Table 3:** Respondents' level of knowledge about COVID-19 (*N* = 712).

**Questions**	**Response**	**Frequency (%)**	**Correct/incorrect**	**Frequency (%)**
K1: The most common symptoms of COVID-19 are fever, cough, fatigue, and shortness of breath.	True	692 (97.2)	Correct	692 (97.2)
	False	6 (0.8)	Incorrect	20 (2.8)
	I don't know	14 (2)		
K2: Unlike the common cold, stuffy nose, runny nose, and sneezing are less common in persons infected with the COVID-19 virus.	True	484 (68)	Correct	484 (68)
	False	171 (24)	Incorrect	228 (32)
	I don't know	57 (8)		
K3: There is currently no certified treatment to cure from COVID-2019.	True	590 (82.9)	Correct^*^	590 (82.9)
	False	29 (4.1)	Incorrect	122 (17.1)
	I don't know	93 (13.1)		
K4: There is currently no available vaccine for prevention from COVID-2019.	True	582 (81.7)	Correct	582 (81.7)
	False	38 (5.3)	Incorrect	130 (18.3)
	I don't know	92 (12.9)		
K5: Elderly and those who have chronic illnesses are more likely to develop to severe cases.	True	687 (96.5)	Correct	687 (96.5)
	False	12 (1.7)	Incorrect	25 (3.5)
	I don't know	13 (1.8)		
K6: Isolation and treatment of people who are infected with the COVID-19 virus are effective ways to reduce the spread of the virus.	True	699 (98.2)	Correct	699 (98.2)
	False	5 (0.7)	Incorrect	13 (1.8)
	I don't know	8 (1.1)		
K7: To prevent the infection by COVID-19, individuals should avoid going to crowded places such as avoiding taking public transportations.	True	698 (98)	Correct	698 (98)
	False	7 (1)	Incorrect	14 (2)
	I don't know	7 (1)		
K8: Persons with COVID-2019 cannot infect the virus to others when a symptom is not present.	True	119 (16.7)	Correct	492 (69.1)
	False	492 (69.1)	Incorrect	220 (30.9)
	I don't know	101 (14.2)		
K9: The COVID-19 virus spreads *via* respiratory droplets of infected individuals during cough or sneezing.	True	667 (93.7)	Correct	667 (93.7)
	False	29 (4.1)	Incorrect	45 (6.3)
	I don't know	16 (2.2)		
K10: It is not necessary for children and young adults to take measures to prevent the infection by the COVID-19 virus.	True	25 (3.5)	Correct	666 (93.5)
	False	666 (93.5)	Incorrect	46 (6.5)
	I don't know	21 (2.9)		
K11: People who have contact with someone infected with the COVID-19 virus should be isolated for 14 days to ensure they are not infected.	True	705 (99)	Correct	705 (99)
	False	1 (0.1)	Incorrect	7 (1)
	I don't know	6 (0.8)		
K12: People who do not have COVID-19 can wear face masks as a protection from getting the virus	True	554 (77.8)	Correct	554 (77.8)
	False	134 (18.8)	Incorrect	158 (22.2)
	I don't know	24 (3.4)		

*K, knowledge. Knowledge was assessed by giving 1 to a correct answer, 0 to an incorrect answer. A score >10 was taken as good knowledge while ≤ 10 as poor knowledge*.

* *By the time of data collection period (April 30 to July 2), there was no specific treatment approved for the disease*.

### Respondents' Attitude Toward COVID-19

Of the 712 respondents, 702 (98.6%) showed a positive attitude (0.84 ± 0.09), while 10 (1.4%) displayed a negative attitude (0.27 ± 0.16) toward COVID-19. The mean score of the group's attitude toward the disease was 0.83 ± 0.11. The majority of the respondents (*n* = 670, 94.1%) agreed that COVID-19 is preventable, will eventually be successfully controlled (*n* = 587, 82.4%), and that the Kingdom of Saudi Arabia will be able to eradicate the new epidemic (*n* = 685, 96.2%) (Table 4).

**Table 4:** Distribution of participant response to attitude related questions on COVID-19 (*N* = 712).

**Questions**	**Answer**	**Frequency (%)**
A1: COVID-19 infection is preventable.	Agree	670 (94.1)
	Uncertain	32 (4.5)
	Disagree	10 (1.4)
A2: COVID-19 will finally be successfully controlled?	Agree	587 (82.4)
	Uncertain	102 (14.3)
	Disagree	23 (3.2)
A3: With the God will, the Kingdom of Saudi Arabia will be able to eradicate the new epidemic of Corona.	Agree	685 (96.2)
	Uncertain	23 (3.2)
	Disagree	4 (0.6)
A4: Wearing a face mask during working hours.	Agree	635 (89.2)
	Uncertain	22 (3.1)
	Disagree	55 (7.7)
A5: Handling a COVID-19 patient threatens security and safety workers.	Agree	573 (80.5)
	Uncertain	55 (7.7)
	Disagree	84 (11.8)
A6: Closure of schools and workplaces during COVID-19 pandemic as a preventive measure.	Agree	702 (98.6)
	Uncertain	7 (1)
	Disagree	3 (0.4)
A7: Implementation of preventive measures (lockdown, social distancing, and others) can stop the spread of COVID-19.	Agree	701 (98.5)
	Uncertain	8 (1.1)
	Disagree	3 (0.4)
A8: In fighting COVID-19, everyone has the same shares of responsibility.	Agree	681 (95.6)
	Uncertain	12 (1.7)
	Disagree	19 (2.7)

*A, Attitude was assessed by giving 1 to Agree and 0 to Uncertain or Disagree. A score ≥0.5 was taken as positive and <0.5 as negative attitude. Mean attitude score was 0.83 ± 0.11. Mean attitude score ± SD: A1 0.94 ± 0.24, A2 0.82 ± 0.38, A3 0.96 ± 0.19, A4 0.89 ± 0.31, A5 0.12 ± 0.32, A6 0.99 ± 0.12, A7 0.98 ± 0.12, A8 0.96 ± 0.20*.

Multiple logistic regression analysis revealed a significant association between the respondents and chronic disease (vs. others, OR: 0.354, *P* = 0.001) with the disagreement that COVID-19 infection is preventable. Female gender (OR: 0.693; *P* = 0.029) and age group of 35–49 years vs. 50+ years were significantly linked (OR: 0.694; *P* = 0.004) to the disagreement that COVID-19 will eventually be successfully controlled (Table 5). The attitude toward the ability of Saudi Arabia to eradicate the new epidemic differed across the categories of marital status (married vs. others, OR 0.421; *P* = 0.027). Accordingly, childless respondents were 2.5 times (OR: 2.512; *P* = 0.020) more likely to display a negative attitude (A3) as compared to those with children (Table 5). Furthermore, while the majority of the respondents agreed to wearing face masks during working hours (*n* = 635, 89.2%), the 35–49 year age group (vs. 50+ years) were inclined to disagree (OR: 0.549; *P* = 0.041) with doing so. The most noticeable negative attitude was expressed on the question of whether or not the handling of COVID-19 patients threatened them (0.12 ± 0.32). This differed significantly between genders (female vs. male, OR: 0.916, *P* = 0.002) (Table 5).

**Table 5:** Factors affecting good attitude among the participants toward COVID-19.

	OR (CI)^*^	*P*-value^*^
**A1. Disagree that COVID-19 infection is preventable (vs. agree or uncertain)**		
Gender (Female vs. male)	0.854 (0.475–1.536)	0.634
Marital status (Married vs. others)	0.977 (0.510–1.869)	1.000
Education level (high school degree and below vs. Bachelor's degree or above)	1.881 (0.961–3.681)	0.073
Age (20–34) vs. 50 and above	1.662 (0.694–3.980)	0.286
Age (35–49) vs. 50 and above	0.564 (0.225–1.414)	0.247
Chronic disease (no vs. yes)	0.354 (0.196–0.636)	0.001
Children (no vs. yes)	1.407 (0.749–2.646)	0.352
Years of experience (less or equal than 10 years vs. more than 10 years)	1.200 (0.635–2.266)	0.621
**A2. Disagree that COVID-19 will finally be successfully controlled (vs. agree or uncertain)**		
Gender (Female vs. male)	0.693 (0.503–0.955)	0.029
Marital status (Married vs. others)	1.088 (0.758–1.565)	0.365
Education level (high school degree and below vs. Bachelor's degree or above)	1.367 (0.971–1.925)	0.071
Age (20–34) vs. 50 and above	0.694 (0.449–1.071)	0.130
Age (35–49) vs. 50 and above	0.529 (0.354–0.790)	0.004
Chronic disease (no vs. yes)	0.915 (0.620–1.352)	0.709
Children (no vs. yes)	0.991 (0.683–1.439)	1.000
Years of experience (less or equal than 10 years vs. more than 10 years)	0.990 (0.710–1.381)	1.000
**A3. Disagree that the Kingdom of Saudi Arabia will be able to eradicate the new epidemic of Corona (vs. agree or uncertain)**		
Gender (Female vs. male)	0.587 (0.276–1.247)	0.173
Marital status (Married vs. others)	0.421 (0.201–0.879)	0.027
Education level (high school degree and below vs. Bachelor's degree or above)	1.335 (0.608–2.930)	0.551
Age (20–34) vs. 50 and above	1.234 (0.453–3.361)	0.798
Age (35–49) vs. 50 and above	0.404 (0.139–1.183)	0.151
Chronic disease (no vs. yes)	0.687 (0.296–1.592)	0.454
Children (no vs. yes)	2.512 (1.199–5.261)	0.020
Years of experience (less or equal than 10 years vs. more than 10 years)	1.076 (0.491–2.359)	1.000
**A4. Disagree with Wearing face mask during working hours (vs. agree or uncertain)**		
Gender (Female vs. male)	0.790 (0.518–1.205)	0.279
Marital status (Married vs. others)	1.113 (0.687–1.804)	0.788
Education level (high school degree and below vs. Bachelor's degree or above)	1.309 (0.837–2.049)	0.268
Age (20–34) vs. 50 and above	0.653 (0.360–1.184)	0.193
Age (35–49) vs. 50 and above	0.549 (0.321–0.940)	0.041
Chronic disease (no vs. yes)	0.564 (0.359–0.886)	0.21
Children (no vs. yes)	1.256 (0.790–1.997)	0.327
Years of experience (less or equal than 10 years vs. more than 10 years)	0.717 (0.469–1.096)	0.130
**A5. Disagree that Handling COVID-19 patient threatens security and safety workers (vs. agree or uncertain)**		
Gender (Female vs. male)	0.916 (0.869–0.966)	0.002
Marital status (Married vs. others)	1.061 (0.993–1.134)	0.070
Education level (high school degree and below vs. Bachelor's degree or above)	1.016 (0.961–1.074)	0.636
Age (20–34) vs. 50 and above	0.944 (0.873–1.021)	0.231
Age (35–49) vs. 50 and above	0.946 (0.884–1.012)	0.211
Chronic disease (no vs. yes)	0.987 (0.924–1.054)	0.771
Children (no vs. yes)	0.977 (0.914–1.043)	0.497
Years of experience (less or equal than 10 years vs. more than 10 years)	0.963 (0.913–1.016)	0.223
**A6. Disagree with Closure of schools and workplaces during COVID-19 pandemic as preventive measure (vs. agree or uncertain)**		
Gender (Female vs. male)	0.366 (0.095–1.404)	0.200
Marital status (Married vs. others)	0.391 (0.114–1.335)	0.154
Education level (high school degree and below vs. Bachelor's degree or above)	NA (infinite)	0.007
Age (20–34) vs. 50 and above	0.712 (0.121–4.190)	0.658
Age (35–49) vs. 50 and above	0.451 (0.084–2.425)	0.305
Chronic disease (no vs. yes)	0.561 (0.147–2.142)	0.417
Children (no vs. yes)	4.709 (1.345–16.494)	0.016
Years of experience (less or equal than 10 years vs. more than 10 years)	0.807 (0.230–2.832)	0.746
**A7. Disagree with Implementation of preventive measures (lockdown, social distancing, and others) can stop the spread of COVID-19 (vs. agree or uncertain)**		
Gender (Female vs. male)	0.320 (0.086–1.197)	0.124
Marital status (Married vs. others)	0.326 (0.100–1.055)	0.083
Education level (high school degree and below vs. Bachelor's degree or above)	NA (infinite)	0.004
Age (20–34) vs. 50 and above	NA (infinite)	0.309
Age (35–49) vs. 50 and above	NA (infinite)	0.359
Chronic disease (no vs. yes)	0.421 (0.125–1.417)	0.238
Children (no vs. yes)	3.767 (1.164–12.192)	0.028
Years of experience (less or equal than 10 years vs. more than 10 years)	0.941 (0.278–3.184)	1.000
**A8. Disagree with In fighting COVID-19, everyone has the same and shares the same responsibility (vs. agree or uncertain)**		
Gender (Female vs. male)	0.911 (0.458–1.814)	0.855
Marital status (Married vs. others)	0.282 (0.141–0.565)	0.000
Education level (high school degree and below vs. Bachelor's degree or above)	1.919 (0.871–4.230)	0.133
Age (20–34) vs. 50 and above	4.035 (0.952–17.108)	0.042
Age (35–49) vs. 50 and above	1.353 (0.308–5.942)	1.000
Chronic disease (no vs. yes)	1.002 (0.419–2.394)	1.000
Children (no vs. yes)	3.349 (1.691–6.631)	0.001
Years of experience (less or equal than 10 years vs. more than 10 years)	1.844 (0.806–4.219)	0.178

* *Derived from Fisher exact test and risk assessment. Attitude was assessed by giving 1 to Agree, 0 to Uncertain or Disagree. A score of ≥0.5 was taken as positive attitude while <0.5 as negative attitude. Mean attitude score was 0.83 ± 0.11. Mean Attitude Score ± SD: A1 0.94 ± 0.24, A2 0.82 ± 0.38, A3 0.96 ± 0.19, A4 0.89 ± 0.31, A5 0.12 ± 0.32, A6 0.99 ± 0.12, A7 0.98 ± 0.12, A8 0.96 ± 0.20. A, Attitude, PA, Positive attitude, NA, Negative Attitude*.

Nearly all of the respondents agreed on the closure of schools and workplaces during the COVID-19 pandemic as a preventive measure and that the implementation of such measures could stop the spread of the disease (*n* = 702; 98.6%, *n* = 701; 98.5%, respectively). These attitudes differed significantly across the educational levels and parenthood status. Childless respondents were 4.7 times (OR: 4.709; *P* = 0.016) more likely to express a negative attitude toward school closure (A6) and 3.8 times (OR: 3.767; *P* = 0.028) more likely to disagree with the implementation of preventative measures (A7) as compared to those with children (Table 5).

In total, 681 of the participants (95.6%) agreed with the notion of everyone bearing the same responsibility in fighting COVID-19. However, this notion varied significantly across the marital categories, age, and family status. Respondents aged 20–34 years were four times (OR = 4.035; *P* = 0.042) more likely to have a negative attitude than those aged 50 and above, while respondents with no children were 3.3 times (OR = 3.349; *P* = 0.001) more likely to show a negative attitude compared to those with children (Table 5).

### Respondents' Level of Practice Toward COVID-19

The vast majority of the participants used soap and water to wash their hands continuously (696, 97.8%), covered their nose and mouth in sneezing or coughing (701, 98.5%), disposed of used tissues (703, 98.7%), and avoided crowded places (697, 97.9%). Also, most of the participants used face masks upon leaving their home (594, 83.4%) and maintained healthy eating (601, 84.4%) and lifestyles (602, 84.6%) (Table 6).

**Table 6:** Frequency distribution of participant's response on practice related questions on COVID-19 (*N* = 712).

**Questions**	**Answers**	**Frequency (%)**
P1: Use soap and water to wash my hands continuously.	Yes	696 (97.8)
	No	2 (0.3)
	Sometimes	14 (2)
P2: Cover my nose and mouth during sneezing or coughing.	Yes	701 (98.5)
	No	4 (0.6)
	Sometimes	7 (1)
P3: Throw away the used tissue	Yes	703 (98.7)
	No	2 (0.3)
	Sometimes	7 (1)
P4: Use face mask when leaving home.	Yes	594 (83.4)
	No	49 (6.9)
	Sometimes	69 (9.7)
P5: Keep on healthy eating (like eating food emphasizes with fruits, vegetables, and avoid carbohydrate and non-essential fat).	Yes	601 (84.4)
	No	26 (3.7)
	Sometimes	85 (11.9)
P6: Keep on healthy lifestyles (like training and early sleep)	Yes	602 (84.6)
	No	34 (4.8)
	Sometimes	76 (10.7)
P7: Avoid crowded places	Yes	697 (97.9)
	No	5 (0.7)
	Sometimes	10 (1.4)

*P, Practice*.

### Factors Influencing the Knowledge, Attitude, and Practice Scores

The association of the participants' demographic characteristics with the knowledge, attitude, and practice scores is displayed in Table 7. Accordingly, age and educational levels were significantly associated with a higher knowledge and attitude scores. Thus, Bachelor's degree holders or above showed a greater knowledge (10.76 ± 1.34 vs. 10.42 ± 1.47; *p* = 0.002) and positive attitude (0.85 ± 0.10 vs. 0.82 ± 0.12; *p* = 0.001) toward COVID-19 than high school certificate holders and below. In addition, the 50–64-year-old age group exhibited a greater knowledge (10.69 ± 1.57 vs. 10.32 ± 0.62 and 10.63 ± 1.28; *p* = 0.023), while the 35–49-year-old age group showed a more positive attitude (0.85 ± 0.09 vs. 0.82 ± 0.15 and 0.81 ± 0.12, p = 0.001) compared to others. It was also revealed that females (0.85 ± 0.10 vs. 0.82 ± 0.13; *P* = 0.001), individuals free from chronic disease (0.84 ± 0.11 vs. 0.81 ± 0.12, *P* = 0.018), those residing outside Riyadh (0.85 ± 0.10 vs. 0.83 ± 0.12; *p* = 0.009), or those who were not employed in a health facility (0.84 ± 0.12 vs. 0.81 ± 0.11; *p* = 0.014) showed a more positive attitude than their counterparts. Similarly, females (1.92 ± 0.16 vs. 1.87 ± 0.23; *p* = 0.000), those living outside Riyadh (1.93 ± 0.14 vs. 1.88 ± 0.22; *p* = 0.000), or those working in the private sector (1.92 ± 0.17 vs. 1.89 ± 0.21; *p* = 0.035) had better practices than their counterparts. Additionally, total knowledge scores varied significantly across age groups, categories of marital and parenthood status, and workplace (private vs. government sectors). Married individuals (10.63 ± 1.28 vs. 10.36 ± 1.74, *p* = 0.040), parents (10.63 ± 1.34 vs. 10.33 ± 1.65; *p* = 0.029), and government employees (10.66 ± 1.30 vs. 10.38 ± 1.62; *p* = 0.018) were better informed than their counterparts. Thus, the Spearman correlation test revealed a significant positive relationship between knowledge and both attitude (*r* = 0.182, *p* = 0.000) and practice (*r* = 0.186; *p* = 0.022) of security and safety workers toward COVID-19.

**Table 7:** Influence of respondent characteristics on their level of knowledge, attitude, and practice toward COVID-19 (*n* = 712).

**Characteristics**	**Frequency (%)**	**Knowledge score (mean ± SD)**	*P*-value^*^	**Attitude score (mean ± SD)**	*P*-value^*^	**Practice score (mean ± SD)**	*P*-value^*^
**Gender**							
Male	328 (46.1)	10.53 ± 1.61	0.658	0.82 ± 0.13	0.001	1.87 ± 0.23	0.000
Female	384 (53.9)	10.58 ± 1.26		0.85 ± 0.10		1.92 ± 0.16	
**Marital status**							
Married	512 (71.9)	10.63 ± 1.28	0.040	0.84 ± 0.10	0.210	1.90 ± 0.19	0.944
Others	200 (28.1)	10.36 ± 1.74		0.81 ± 0.17		1.90 ± 0.22	
**Children**							
No children	172 (24.1)	10.33 ± 1.65	0.029	0.82 ± 0.16	0.073	1.88 ± 0.23	0.272
Has children	540 (75.8)	10.63 ± 1.34		0.84 ± 0.10		1.90 ± 0.19	
**Educational level**							
High school degree and below	427 (60.0)	10.42 ± 1.47	0.002	0.82 ± 0.12	0.001	1.90 ± 0.18	0.619
Bachelor's degree or above	285 (40.0)	10.76 ± 1.34		0.85 ± 0.10		1.89 ± 0.22	
**Age**							
20–34	198 (27.9)	10.32 ± 1.62	0.023	0.82 ± 0.15	0.001	1.89 ± 0.21	0.763
35–49	417 (58.8)	10.63 ± 1.28		0.85 ± 0.09		1.90 ± 0.20	
50–64	94 (13.2)	10.69 ± 1.57		0.81 ± 0.12		1.90 ± 0.16	
**Do you have chronic disease**							
No	574 (80.6)	10.57 ± 1.40	0.476	0.84 ± 0.11	0.018	1.90 ± 0.19	0.088
Yes	138 (19.38)	10.48 ± 1.53		0.81 ± 0.12		1.87 ± 0.22	
**Region**							
Riyadh	512 (71.9)	10.53 ± 1.46	0.493	0.83 ± 0.12	0.009	1.88 ± 0.22	0.000
Others	200 (28.1)	10.62 ± 1.35		0.85 ± 0.10		1.93 ± 0.14	
**Workplace**							
Health facility	150	10.61 ± 1.25	0.627	0.81 ± 0.11	0.014	1.89 ± 0.18	0.638
Others	562	10.54 ± 1.47		0.84 ± 0.12		1.90 ± 0.20	
**You work in**							
Private Sector	261 (36.6)	10.38 ± 1.62	0.018	0.83 ± 0.14	0.306	1.92 ± 0.17	0.035
Government Sector	451 (63.3)	10.66 ± 1.30		0.84 ± 0.10		1.89 ± 0.21	
**Years of experience**							
<10 years	463 (65)	10.54 ± 1.40	0.609	0.83 ± 0.12	0.585	1.90 ± 0.20	0.575
More than 10 years	249 (35)	10.59 ± 1.50		0.83 ± 0.10		1.89 ± 0.19	
**The nature of your work**							
Field work	623 (87.5)	10.56 ± 1.45	0.968	0.83 ± 0.12	0.915	1.90 ± 0.20	0.807
Administrative work	89 (12.5)	10.56 ± 1.28		0.83 ± 0.09		1.89 ± 0.22	
**Were you on duty from February 2020 to May 2020 (at the time of the onset of COVID-19)**							
No	75 (10.5)	10.63 ± 1.30	0.652	0.84 ± 0.14	0.415	1.91 ± 0.14	0.434
Yes	637 (89.4)	10.55 ± 1.44		0.83 ± 0.11		1.90 ± 0.20	

* *Derived from T-test, ANOVA. Knowledge was assessed by giving 1 to a correct answer and 0 to an incorrect answer. A score >10 was taken as good knowledge while ≤ 10 as poor knowledge. Mean knowledge score was 10.57 ± 1.43. Attitude was assessed by giving 1 to Agree and 0 to Uncertain or Disagree. Score of ≥0.5 was taken as positive attitude while <0.5 as negative attitude. Mean attitude score was 0.83 ± 0.11. Practice was assessed by giving 2 to Yes, 1 to Sometimes, 0 to No. Score of ≥ was taken as good practice, while <1 as poor practice. Mean practice score was 1.90 ± 0.20*.

## Discussion

In the course of the rapidly rising COVID-19 cases, security and safety workers carrying out unusual tasks are continually engaged in combating the disease without any specific preparation. Indeed, they are performing an enormous task in supporting both healthy and infected citizens, possibly impacting their own health during and after the accomplishment of their responsibilities. Effective prevention and control of COVID-19 is attainable through enhancing the KAP of the population toward the disease. Appropriate knowledge is crucial in order to embrace better attitudes and in adopting precautionary practices to prevent and control the spread of the disease (20, 21). Hence, this study aimed to assess the KAP of security and safety workers toward COVID-19 in Saudi Arabia. Our findings indicate that most respondents were sufficiently knowledgeable about the disease, as the majority of them answered the knowledge questions correctly providing an overall 88.0% correct rate on these questions. To the best of our knowledge, this study is the first of its kind not only in Saudi Arabia, but also in the Gulf Cooperation Council (GCC) countries as a whole.

Studies in various parts of the world have arrived at partly different conclusions on the same or similar subject, possibly due to a couple of reasons. For example, the knowledge score attained in our study is slightly lower than the 90% reported in the Chinese general population (7), but somewhat higher than the KAP reported toward COVID-19 in several local and other international studies (15, 18, 22–25). Besides, a cross-sectional study recently reported a greater knowledgeability about COVID-19 in Saudi Arabia (22), while another pointed to a moderate general awareness level (58%) toward this emerging disease among Saudi communities in Riyadh (23). Yet another investigation demonstrated a moderate knowledge level (55.0%) on COVID-19 transmission and preventive measures in the general population in Saudi Arabia (24). In contrast, a cross-sectional study in Uganda described a lack of knowledge among certain population groups (drivers and security agents) on the prevention of the spread of the disease (26). Also, Sarfaraz et al. (27) assessed the knowledge and attitude of dental health practitioners from 23 different countries across the world, indicating a lack of knowledge (4.19 ± 1.88 out of 12) in dentists about the fundamental aspects of disinfection protocols. Moreover, a multinational cross-sectional study conducted in three Middle Eastern countries (Jordan, Saudi Arabia, and Kuwait) to explore the knowledge and practices of this population toward the disease reported a relatively low level of knowledge, particularly on its transmission routes (25). These variations might be ascribed to differences in the socioeconomic status of the study participants. However, inconsistences in the measurement and scoring systems may also hamper accurate comparisons of knowledge levels among different studies. Significant predictors for knowledgeability included educational level, age, marital, and parenthood status, as well as the employment sector (private or government) of the participants. Thereby, in concordance with observations by several other studies (7, 15, 18, 22), Bachelor's degree holders or higher appeared to be better informed. Furthermore, older and married individuals, as well as those with children or working in a government institution attained higher knowledge scores when compared to their counterparts. Conversely, lack of knowledge contributed to the emergence and spread of the outbreak potentially increasing the burden of the disease on the community. Establishment of factors associated with knowledge gaps among participants should be valuable for policymakers to recognize target populations for health educational activities in the outbreak.

Our study revealed an overall positive attitude by safety and security workers toward COVID-19 as a preventable disease as indicated by the fact that the majority of them agreed on this notion and were convinced of it being eventually successfully controlled. The confidence of the participants on the ability of Saudi Arabia to eradicate this new epidemic was consistent with the findings from other studies conducted in Saudi Arabia (22), China (7), and Malaysia (18), supporting the confidence on its curability and their respective countries succeeding in the battle against the pandemic. In our study, the positive attitude and high confidence level of the participants were probably built on the previous experience of the Saudi government in response to the Middle East Respiratory Syndrome (MERS) epidemic in 2012 (28), which helped the country reinstate the improved public health alerting system and infection control policies. Thus, the experience gathered from combating MERS placed Saudi Arabia on a high sense of alert and readiness to take instant action and drastic measures to curb the spread of COVID-19 (29). In line with this, the country imposed a number of extreme measures such as enforced lockdown, implementing curfews, stopping all flights (domestic and international), suspending Umrah, interdiction of social and religious gatherings, restricting outdoor activities, closure of mosques, and suspension of schools and universities eventual shifting to remote learning and virtual classrooms.

In the current study, the majority of the safety and security workers were following good and safe COVID-19 prevention practices. Most participants reported taking precautions such as avoiding crowded places, practicing proper hand hygiene, covering their nose and mouth when sneezing or coughing, and wearing face masks on leaving their homes. These are very vital practices to prevent the person to person transfer of the disease. In addition, females and respondents residing outside Riyadh or employed in private sectors had better practices as compared to their male counterparts. The findings related to prevention practices are consistent with those from other studies such as that of Alahdal et al. (23) in Saudi Arabia, Azlan et al. (18) in Malaysia, Olum et al. (15) in Uganda, Zhong et al. (7) in China, Abdel Wahed et al. (30) in Egypt, and Almofada et al. (31) and Al-Hanawi et al. (22) in Saudi Arabia. These preventive practices are attributable to the educational materials provided by the MOH and WHO through multiple media platforms for the detection, prevention, and control of COVID-19. It is important to focus on providing precise knowledge to individuals as this has a significant influence on the attitudes and practices in a pandemic. In this study, a higher knowledge level was displayed in both the attitude and practice of the participants as demonstrated by the Spearman correlation test showing a significant positive relationship between knowledge as well as both the attitude and practices of security and safety workers toward COVID-19.

Lastly, the information on COVID-19 used by most of the participants in our study was acquired primarily through television, followed by internet and social media. This is concordant with previous studies pointing similarly to TV and/or radio (32) and social media (33) as the primary sources of information, therefore highlighting the important roles for these platforms in spreading knowledge. However, while social media and internet platforms provide an easy accessibility to information, they can also be a source of misinformation. Currently, a vast diversity of information is readily available through the internet, including unverified malicious information that can spread quickly and misguide individuals, hence causing fear and anxiety among the population. People with anxiety may become panicked and are more likely to make mistakes leading to irrational behavior. Likewise, a cross-sectional study among dental professionals in 30 different countries across the globe reported that dentists were in a state of anxiety of getting infected and fear of carrying infections from their practices to their families while working during the current viral outbreak (34). On the other hand, it can be assumed that a clear communication and an updated educational content provided by the MOH about COVID-19 through multiple media platforms probably contributed to improving public knowledge and preparedness during the current pandemic (31).

This study has some limitations. These include the convenient sampling and self-reported questionnaires which partly depended on participant honesty and recall ability which may therefore be subject to a recall bias. Moreover, our study was designed as a cross-sectional survey, thereby limiting our ability to identify causality between study variables. In addition, there is yet a lack of studies assessing the KAP of safety and security workers during the COVID-19 pandemic worldwide, which limited our ability to compare our findings with similar groups. Accordingly, we suggest cohort studies in future to validate our findings.

## Conclusion

Our study suggests that most of the safety and security workers possessed sufficient knowledge and positive attitude as well as exercised appropriate practices toward COVID-19. The significant predictors for the positive attitude were gender, educational level, age, and workplace similar to those found in previous studies. Also, in this study, a better knowledge level was reflected in both the attitude and practices of the participants. This finding demonstrates that virtuous knowledge is important in empowering individuals to demonstrate better attitudes and practices for current pandemic risk reduction as well as for future epidemics.

## Data Availability Statement

The raw data supporting the conclusions of this article will be made available by the authors, without undue reservation.

## Ethics Statement

The studies involving human participants were reviewed and approved by King Saud University College of Medicine Institutional Review Board. The patients/participants provided their written informed consent to participate in this study.

## Author Contributions

MA, AA, HA, and GA conceptualized the study. HA and GA collected data. MA analyzed and interpreted the data. MA and FK wrote the first draft. AA, FSA, FYA, and GS edited the draft. MA and FK reviewed and edited the final manuscript. All authors approved the final version.

## Conflict of Interest

The authors declare that the research was conducted in the absence of any commercial or financial relationships that could be construed as a potential conflict of interest.
